# Genetic diversity leads to differential inflammatory responses to cigarette smoke in mice

**DOI:** 10.14814/phy2.70199

**Published:** 2025-01-22

**Authors:** Md Imam Faizan, Gagandeep Kaur, Sadiya Bi Shaikh, Felix Effah, Hoshang Unwalla, Irfan Rahman

**Affiliations:** ^1^ Department of Environmental Medicine University of Rochester Medical Center Rochester New York USA; ^2^ Department of Cellular and Molecular Medicine, Herbert Wertheim College of Medicine Florida International University Miami Florida USA

**Keywords:** cigarette smoke, ETS, genetic diverse strains, inflammation, J:DO, UNC

## Abstract

The use of genetically diverse mouse models offers a more accurate reflection of human genetic variability, improving the translatability of findings to heterogeneous human populations. This approach is particularly valuable in understanding diverse immune responses to disease by environmental exposures. This study investigates the inflammatory responses to acute exposures to mainstream cigarette smoke (CS) and environmental tobacco smoke (ETS) in two genetically diverse mouse strains, CC002/UncJ (UNC) & Diversity Outbred (J:DO). The UM‐HET3 (HET3) mouse strain, typically used in aging intervention studies, has also been used to evaluate the translatability of this model for age‐associated pathologies. The study involves a comprehensive approach, including BALF cytokine analysis, evaluation of lung tissue architecture, assessment of macrophages and its associated proteins (MMP9 & MMP12) abundance. Several cytokines/chemokines were found to be upregulated across three strains. Notably, the UNC strain exclusively showed upregulation of TNF‐α, IL‐17A, and IL‐13, whereas the J:DO showed an upregulation in KC. The number of alveolar macrophages in the lungs of UNC mice was very low at baseline compared to other strains studied in this study, which is indicative of some inherent shift in the pulmonary immune profiles of these inbred mice. In contrast, the J:DO strain, characterized by genetic outbreeding, showed a much more robust lung macrophage response comparable to C57BL/6J. The findings provide valuable insight into how genetic diversity affects immune responses in response to acute CS/ETS exposure, with implications for understanding diverse human responses to environmental stressors in studying lung pathophysiology.

## 1 INTRODUCTION

Over the years, our understanding of the immune system has greatly advanced, mainly due to the extensive use of mouse models in biomedical research. These models offer flexibility due to shorter life spans and the ability to regulate confounders like age, sex, and environment. The use of standard inbred strains (C57BL/6J or BALB/c) in mice has reduced experimental variability, making results more reproducible (Roderick, 2001). However, this genetic uniformity limits the investigation of how genetic diversity influences immunity and disease development in the real‐world (Orrù et al., 2020). As a result, while these models are decisive for controlling experimental variables, they may need to fully capture the genetic and environmental diversity seen in the human population. In contrast, human immunity varies significantly between individuals, driven by factors including but not limited to genetics, environment, sex, age, and prior microbial infections. Understanding these variations is crucial for translating findings from mouse models to the clinic (Zuberi & Lutz, 2016).

The UNC (Strain #:021236) and J:DO (Strain #:009376) mice are recombinant mouse lines developed from eight genetically distinct founder strains: C57BL/6J, A/J, 129S1/SvImJ, NOD/ShiLtJ, NZO/HlLtJ, CAST/EiJ, PWK/PhJ, and WSB/EiJ. Using a funnel breeding strategy, genetic material from all eight founders is integrated into the Collaborative Cross (CC) strains. More than 20 generations of breeding follows to ensure genetic stability and reproducibility within the strains, thus making it a powerful model to study health and disease in the lab setting (Leist & Baric, 2018; Saul et al., 2019; Soni et al., 2024). The J:DO (an outbred strain) represents one of the most genetically diverse mouse populations available, offering a powerful tool for studying human genetic and phenotypic variation, which can also be utilized in a wide range of research applications, including behavioral analysis, toxicological screening and drug resistance (Chesler et al., 2008; Churchill et al., 2004; Churchill et al., 2012; Logan et al., 2013; Svenson et al., 2012). Meanwhile, the UNC mouse (an inbred strain) offers a consistent and reproducible source of genome‐wide genetic variation, making it ideal for complex trait analysis and genetics research (Churchill et al., 2004; Genetics, 2012; Threadgill et al., 2011). This uniform genetic variation allows for deeper exploration of complex traits and facilitates the development of models relevant to human diseases. Research utilizing these mouse strains has already produced significant insights in various fields, including behavioral studies, neurology, immunology, and infectious disease (COVID‐19) research (Hackett et al., 2022; Logan et al., 2013; McMullan et al., 2018; Robertson et al., 2023; Soni et al., 2024).

Multiple studies, including ours, have uncovered various inflammatory responses across different mouse strains (Yao et al., 2008). Still, no study showed the CS‐induced inflammatory response in these genetically diverse mice strains. In addition to the two diverse mouse strains, the UM‐HET3 strain (HET3; Strain #:036603) has gained significant popularity as a well‐established model for studying genetic diversity in aging research (Bou Sleiman et al., 2022; Jayarathne et al., 2022; Mau et al., 2020; Willows et al., 2023). HET3 mice are genetically heterogeneous, produced by crossing two hybrid strains, CByB6F1/J and C3D2F1/J, resulting in a mixed genetic background that more accurately mirrors the genetic diversity found in human populations.

Research focusing solely on single inbred mouse strains, like C57BL/6J or BALB/c, limits our understanding of immunity across genetically diverse populations. Expanding research to include genetically diverse populations, like those provided by UNC/J:DO/HET3 mice, can help identify new models that better reflect human immune responses and disease progression. These diverse mice are a precious tool for studying inflammation and host‐responses to stressors, such as CS/ETS. However, selecting suitable strains for specific research questions can be challenging for broad screening without significant resources.

**FIGURE 1:**
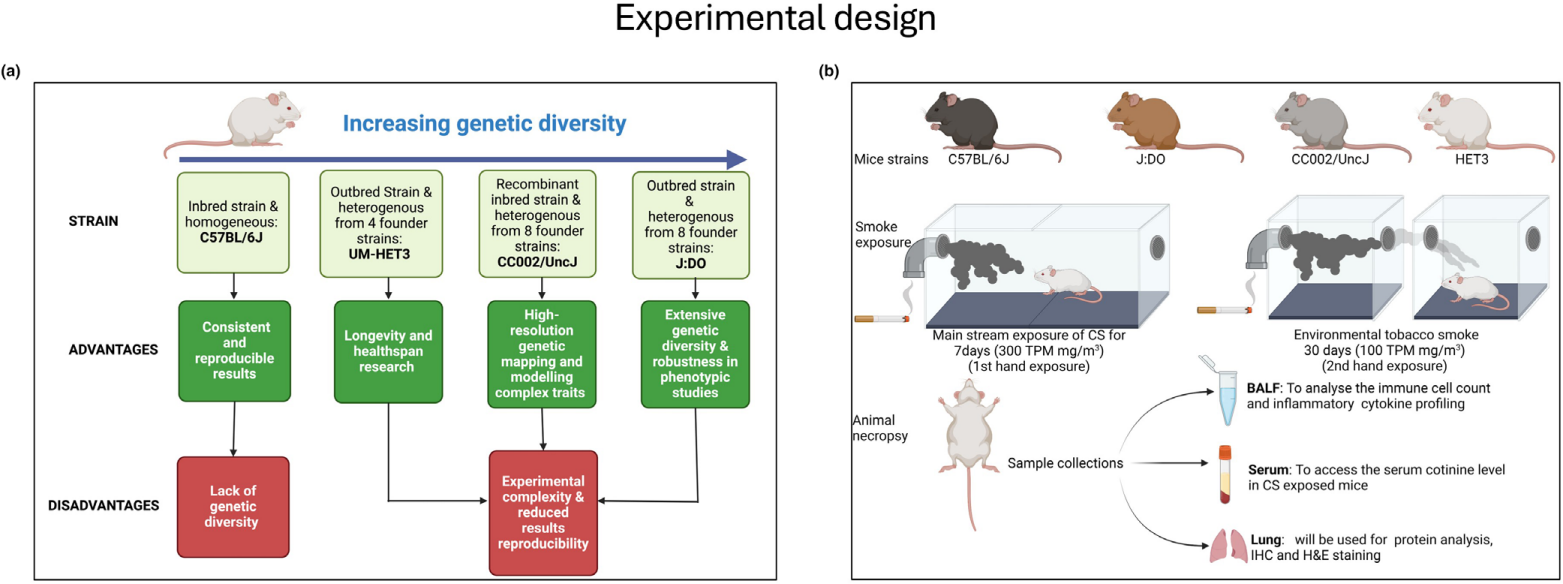
(a) Diagram to elucidate the different strains used in this study: Their use in animal research, and the disadvantages associated with their use. (b) Schematic representation of experimental design for the study using different mice strains. Figures were created with BioRender.com.

The present study highlights a wide range of inflammatory cytokine variations upon CS/ETS exposure across various species. It investigates the pulmonary inflammatory effects of acute CS/ETS exposure in C57BL/6J, UNC, J:DO & HET3 strains (Figure 1a,b). We examined the effects of genetic variations in J:DO, UNC, and C57BL/6J mice by exposing them to 300 mg/m^3^ total particulate matter (TPM) for CS for seven consecutive days and 100 mg/m^3^ TPM for ETS for 30 days. We further investigated the effects of CS and ETS on morphological changes in the lung histology, proinflammatory cytokine release, and differential cell count to study how various mouse strains respond to CS/ETS mediated stress. Understanding the differential susceptibility among these strains will not only enhance our ability to predict and mitigate the adverse health effects of CS/ETS in genetically diverse populations, but it will also help us to choose the best suited mouse model for investigating inflammation and inflammation‐related pathologies.

## 2 MATERIALS AND METHODS

### 2.1 Ethical statements

All the experiments were conducted following the standards and guidelines authorized by the Institutional Biosafety Committee of the University of Rochester. All protocols for housing, handling, exposures, and performing procedures with mice were approved by the University Committee on Animal Research (UCAR) at the University of Rochester (UCAR# UCAR‐2024‐003). Four different mice strains [C57BL/6J (Strain #:000664, RRID:IMSR_JAX:000664), UNC (C002/UncJ; Strain #:021236, RRID:IMSR_JAX:021236), J: DO (Strain #:009376, RRID:IMSR_JAX:009376), and HET3 (Strain #:036603, RRID:IMSR_JAX:036603)] were purchased from Jackson Laboratory. Both male and female mice, aged 2–7 months and weighing between 23 and 26 g, were included in the study. The mice were housed in aseptic condition at University of Rochester's vivarium facility, maintained on a 12‐h light‐dark cycle, with unrestricted access to food and water. Before proceeding with the CS/ETS exposure, the mice were kept for a week in a separate animal room for acclimatization. After the final exposure, the mice were euthanized via IP injection of pentobarbital at a dose of 100 mg/kg body weight according to the approved protocols. Careful efforts were made to apply a thorough and impartial approach during the experimental phase and the analysis of results to ensure reproducibility that meets NIH standards.

### 2.2 Mice model development

Both male and female mice (23–26 g body weight) aged 2–7 months were selected for this study. All mouse strains that is, C57BL/6J, UNC, J:DO and HET3 (*n* = 7–9/group) were obtained from Jackson Laboratory and housed at the University of Rochester Inhalation Facility during the duration of mouse exposures.

### 2.3 Smoke exposure

For mainstream CS exposure, all four strains (C57BL/6J, HET3, UNC, and J:DO) were included and housed in wired cages with individual compartments connected to an aerated plastic chamber. This setup was linked to a Baumgartner–Jaeger system, which delivered controlled smoke [one puff/min with a 2 s duration and 35‐mL volume (2–4 h/day for a total 7 days)] from research‐grade cigarettes (Hwang et al., 2014; Kaur et al., 2021; Kaur et al., 2023; Sundar et al., 2018; Yao & Rahman, 2012). The mainstream smoke was mixed with filtered air before being administered to the mice. A total exposure of 300–400 mg/m^3^ TPM was provided to the mainstream CS group for 7 days, after which the mice were sacrificed, and serum, lung tissue, and bronchoalveolar lavage fluid (BALF) were collected. For ETS exposure, a TPM of 100 mg/m^3^ was administered via Teague machine for 1 month (5 days a week, 5 h/days hours/day), with mice sacrificed after the final exposure (Figure 1b). Mouse exposed to ambient air acted as controls for both CS and ETS exposure. Notably, no significant changes in body weight were observed after the exposures. Temperature, humidity, and CO levels within the exposure chambers were closely monitored using appropriate exposures to minimize the pain and discomfort to the mice. All CS exposures were conducted according to the US Federal Trade Commission protocol, as previously described by our lab (Yao et al., 2008). Mice that were not exposed to CS served as the control (Air) group.

### 2.4 Bronchoalveolar lavage fluid (BALF)

Mice were sacrificed using pentobarbital (100 mg/kg body weight, IP; Abbott Laboratories). The trachea was punctured for BALF collection, and approximately 2 mL of PBS was used to perform lavage three times with a 2 mL syringe. The collected BALF was centrifuged, with the supernatant stored at −80°C for cytokine analysis, while the pellet was resuspended in PBS for total cell counting. Total cell counts were determined using AO/PI staining of collected cells, with readings obtained on a Nexcelom Cellometer (Bioscience). For differential cell counts, approximately 10 k–30 k cells from the PBS suspension were processed, and a total of 500 cells were counted after Cytospin and Diff‐Quick staining. Neutrophil and macrophage counts were performed in a blinded manner. The counted cell percentages were plotted using GraphPad.

### 2.5 Immunohistochemistry (IHC)

The larger lung lobes of exposed and control mice were inflated with 1% low melting agarose and fixed in 10% formalin overnight. The tissues were then embedded in paraffin and sectioned at a thickness of 5 μm. The IHC procedure began with deparaffinization in 100% xylene, followed by dehydration through a graded ethanol series (100%, 95%, 70%, 50%). Antigen retrieval was performed using a citrate buffer (pH = 6) for 15 min in a water bath set at 95°C. Endogenous peroxidase activity was blocked with 3% H₂O₂ in methanol, and sections were permeabilized with 0.4% Triton X‐100 along with 1% goat serum. To prevent non‐specific binding, sections were blocked with 10% goat serum for 30 min, followed by overnight incubation with the primary antibody 1:200 for CD68 (Cat#CST76437, Cell Signaling Technologies, Danvers, MA) at 4°C. The following day, tissue sections were washed three times and incubated with the appropriate secondary antibody (1:250 dilution. Cat#1706515, Bio‐Rad) for an hour. Sections were treated with DAB (Cat#ab64238, Abcam) for 10 min and counterstained with hematoxylin for 1 min. After rinsing and dehydration through graded ethanol (50%, 70%, 95%, 100%), sections were mounted with DPX mountant and visualized under brightfield microscopy at 20× magnification. Minimum of three slides were stained for each group, with 10 images randomly taken from various regions. IHC image analyses were performed using ImageJ. The TIFF IHC images were loaded into the software, where the color deconvolution tool was applied using the H DAB option to separate the DAB stain into the brown channel, representing the target staining. The brown channel was selected, and the threshold was adjusted to precisely highlight the areas of expression. Quantification was then carried out by analyzing the brown‐stained cells to measure CD68 protein expression (Varghese et al., 2014). CD68+ cells were manually counted using the 10 random images from each tissue section and a sample average was calculated for plotting a graph.

### 2.6 Protein preparation and quantification

Approximately 50 mg of lung tissue was homogenized using an electric homogenizer in 300 μL of RIPA buffer (Cat#ab156034, Abcam) supplemented with protease and phosphatase inhibitors (Cat#1861284, Invitrogen). The homogenate was incubated on ice for 30 min and then centrifuged at 15,000 rpm for 15 min at 4°C. The supernatant was collected and transferred to a labeled tube for protein quantification using the BCA assay (Cat#23225, Invitrogen). The quantified protein was then stored at −80°C until further analysis.

### 2.7 ELISA

ELISA was performed on BALF/lung homogenate samples using single plex ELISA kits, as per the manufacturer's protocol. Briefly, the plate was coated with the capture antibody (unless a coated plate was provided with the kit) 1 day before the experiment. On the day of the experiment, the plates were washed with a wash buffer and then blocked for 30 min. Appropriate BALF/ tissue homogenates and standard volumes were added to the respective wells and incubated at room temperature. After the incubation, respective detection antibodies were added to each plate and again incubated for the recommended period. Next, streptavidin‐HRP was added to the plates. Finally, the substrate solution was added for color development. The plate absorbance was measured at 450 nm using Cytation 5 (BioTek, USA), and the protein/cytokine level was determined in each sample. For the tissue homogenates, the protein amount was normalized by the total protein present in the sample. Note: An undiluted BALF samples were used for the analysis of MCP‐1 (Cat# DY479, R&D Systems) and KC (cat # DY453, R&D Systems) ELISA. For MMP‐9 (Cat# MMPT90, R&D Systems) and MMP‐12 (cat# ab246540, Abcam), a 1:50 and 1:2 dilution of 1 μg/μL lung homogenate protein were used, abu respectively.

### 2.8 Proinflammatory cytokine analysis via Luminex

To determine the inflammatory profile of each mouse strain upon CS/ETS exposure, we performed a multiplex assay using a 23‐plex assay kit (Cat# M60009RDPD, Bio‐Rad) as per the manufacturer's protocol. Antibody‐coated magnetic beads were added to a 96‐well plate, followed by addtion of 50 μL of undiluted BALF samples and standards in duplicate, and incubation for 30 min at room temperature with shaking at 850 ± 50 rpm. After incubation, the detection antibody and Streptavidin‐PE solution were added for 30 and 15 min, respectively. The beads were then resuspended in an assay buffer, and detection was performed using a FlexMap3D system, with concentrations determined based on a standard curve generated during the assay.

### 2.9 Statistical analysis

All statistical analyses were performed using GraphPad Prism 10 software (GraphPad Software Inc., San Diego, CA). A Student's *t*‐test was used to assess the significance of a control and treated groups. For comparisons involving multiple groups, one‐way ANOVA was employed, followed by Tukey's post hoc test. A *p*‐value of <0.05 was considered statistically significant. Graphs were plotted using the mean and standard error of the mean (SEM). *p < 0.05, **p < 0.01, ***p < 0.001, ****p < 0.0001.

## 3 RESULTS

### 3.1 The influx of inflammatory cells following exposure to mainstream CS/ETS

**FIGURE 2:**
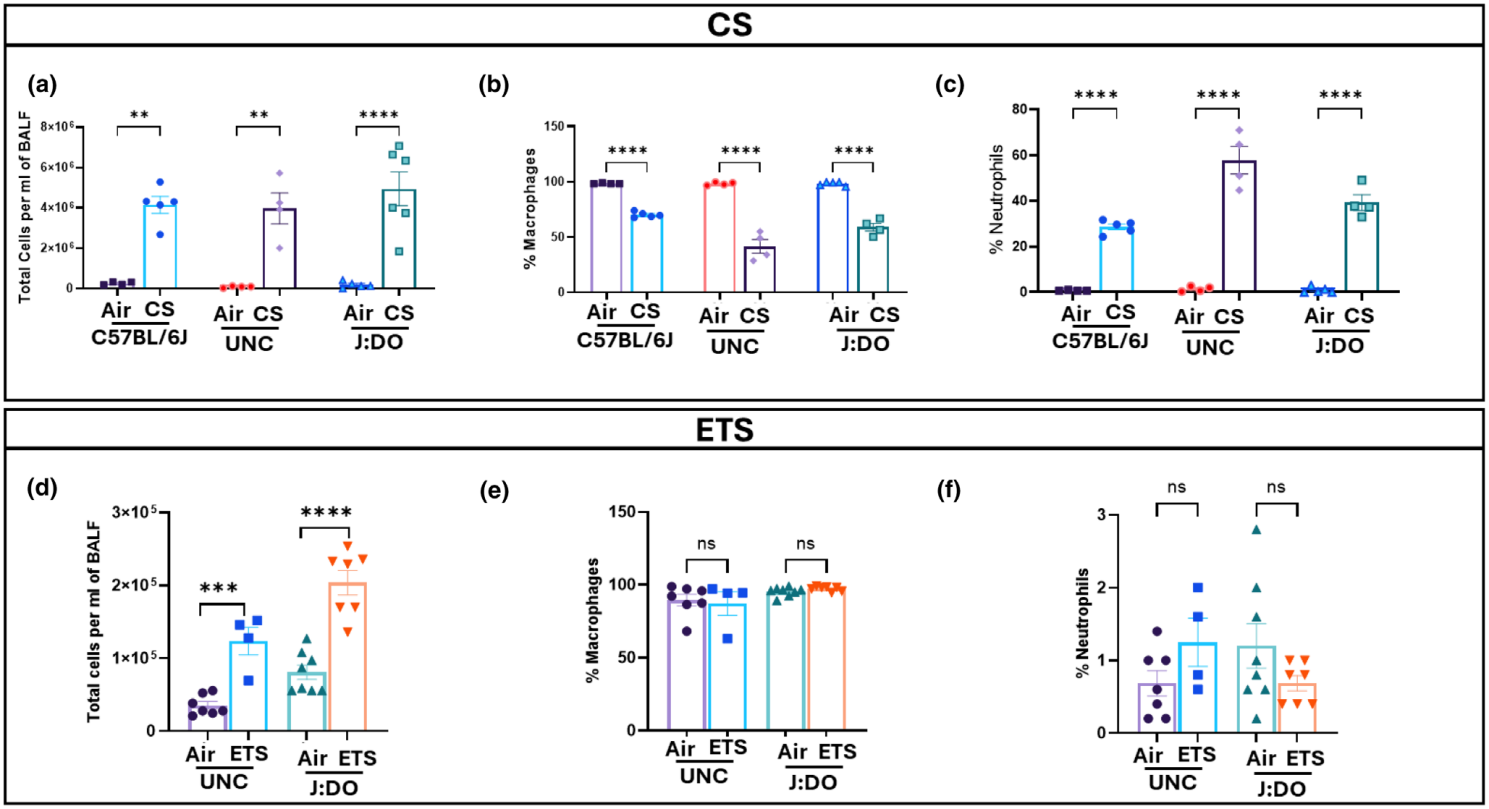
Total cell counts and Immune cell populations (Macrophages & Neutrophils) following exposure to CS and ETS. Following exposure, the mice were lavaged with approximately 2 mL of PBS. One mL of the lavage fluid was used to determine the total cell count, while the remaining 1 mL was utilized for immune cell counts (macrophages and neutrophils). In mainstream the average absolute macrophage count of the total (500 counts) cells were: 351, 493, 208, 491, 295, and 491 counts for C57BL/6J‐CS, C57BL/6J‐AIR, UNC‐CS, UNC‐AIR, J:DO‐CS and J:DO‐AIR respectively and average absolute neutrophil count were: 143, 4, 289, 7, 157, and 5 cells for C57BL/6J‐CS, C57BL/6J‐AIR, UNC‐CS, UNC‐AIR, J:DO‐CS and J:DO‐AIR respectively. In ETS the average absolute macrophage count were: 436, 447, 487, and 476 cells for UNC‐ETS, UNC‐AIR, J:DO‐ETS and J:DO‐AIR respectively and average absolute neutrophil count were: 6, 3, 3, and 6 cells for UNC‐ETS, UNC‐AIR, J:DO‐ETS and J:DO‐AIR respectively. (a, d) Bar graph illustrating total cell counts per mL of BALF, determined using a hemocytometer following AO/PI staining. After Diff‐Quick staining of cytospin slides, approximately 500 immune cells present in the BALF were counted in a blinded manner to determine the percentage of (b, e) macrophages and (c, f) neutrophils. Data are presented as Mean ± SEM from a total of 4–5 mice/group for CS & 4–7 mice/group for ETS exposure. Statistical significance was determined using ordinary one‐way ANOVA for multiple comparisons. Significance levels are indicated as **p < 0.01, ***p < 0.001, and ****p < 0.0001 compared to the air control group.

CS has been extensively studied as a potent inducer of lung inflammation, particularly in mouse models like the C57BL/6J strain (Botelho et al., 2010; Lugade et al., 2014; Pauwels et al., 2011). To better understand the inflammatory response to CS in these genetically diverse mice (UNC and J:DO) along with C57BL/6J, the total cell count was analyzed using AO/PI staining in BALF. The results demonstrated a significant increase in the total cell count in BALF of CS‐exposed mice compared to air controls (Figure 2a). Additionally, to assess the impact of ETS on genetically diverse mice, we also examined the total cell count in ETS‐exposed UNC and J:DO mice, which showed a significant increase in total cell count upon ETS exposure (Figure 2d). This indicates that even a 30‐day acute exposure to ETS (TPM of 100 mg/m^3^) was sufficient to trigger an initial inflammatory response. However, the severity of inflammation was much lower in ETS than that observed in mice exposed to mainstream CS (TPM of 300 mg/m^3^), (Figure 2a,d).

The differential cell counts analysis revealed a significant change in the levels of neutrophils and macrophages upon 7‐day CS exposure in C57BL/6J, UNC, and J:DO mouse strains. The data presented in the graphs reveal a distinct pattern between the air‐control and CS‐exposed groups. Specifically, neutrophil counts in the air group were almost negligible compared to the substantial increase observed in the CS‐exposed groups. At the same time, macrophage levels were notably higher in the air‐control across all strains when compared to the CS‐exposed groups (Figure 2b). Among the strains, UNC mice exhibited the highest percentage of neutrophils in the CS‐exposed group, whereas their macrophage percentages were comparatively lower in the same group. Interestingly, when we conducted the same analysis following ETS exposure, we did not observe any changes in the neutrophil or macrophage percentages. However, ETS exposed UNC mice showed a slight increase in the neutrophil percentages as compared to air controls, whereas the neutrophil numbers seemed to show decreasing trends upon 1‐month ETS exposure in J:DO mice (Figure 2e,f). These findings suggest that neutrophils and macrophages are critical in the inflammatory response to acute CS exposure.

### 3.2 Cytokine and chemokine profiling via Luminex and ELISA

CS has been widely reported in numerous studies to induce the secretion of pro‐inflammatory cytokines, which are easily detectable in BALF (Hodge et al., 2007; Yao et al., 2008). To better understand the effects of CS and ETS on lung inflammation in these genetically diverse mouse strains (UNC & J:DO), we further analyzed the levels of inflammatory cytokines in BALF using a 23‐plex Luminex assay kit. The obtained results revealed a diverse range of cytokines that were differentially regulated across these mice strains following exposure to CS.

**FIGURE 3:**
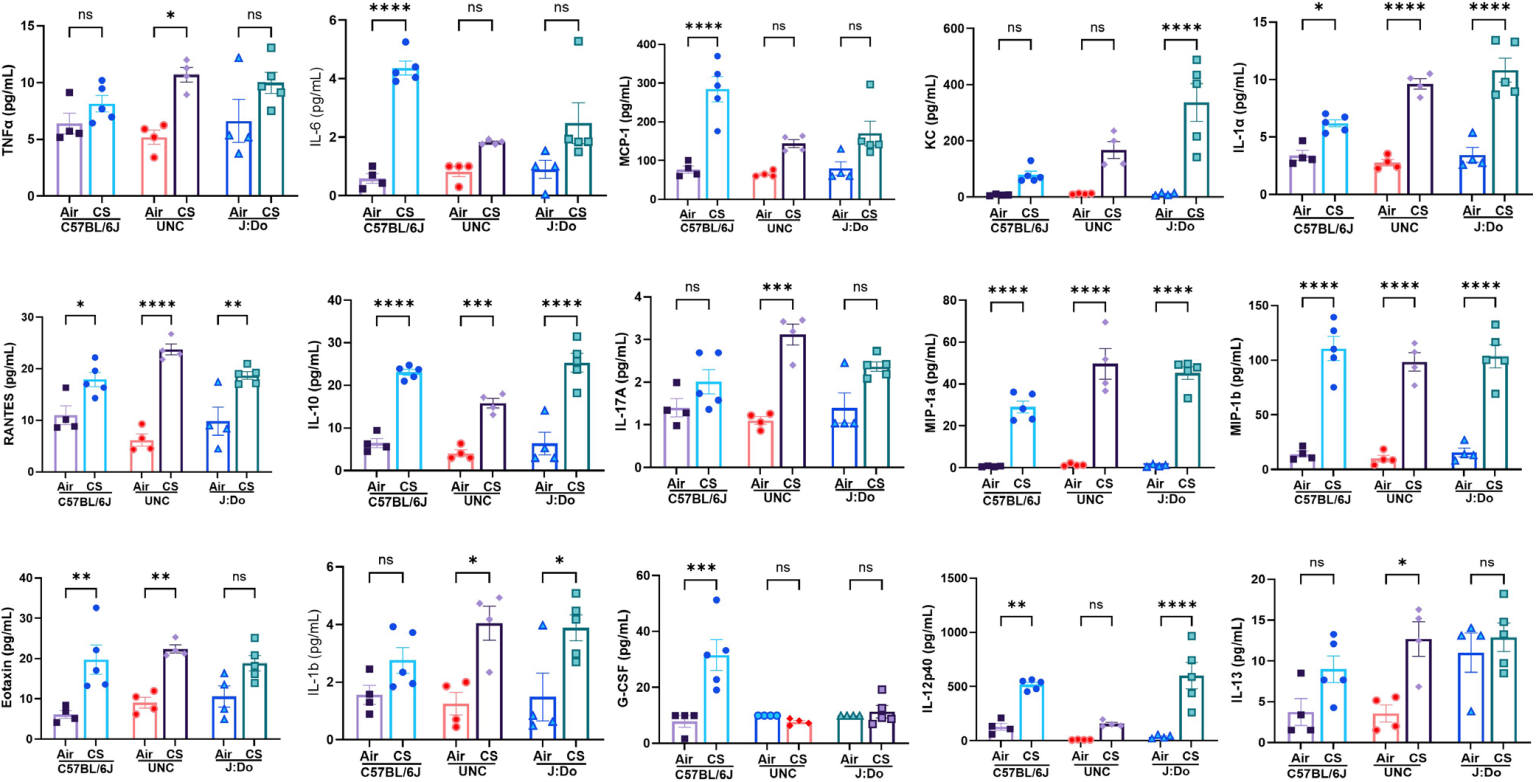
Cytokine analysis in mice strains following CS exposure: The stored BALF samples, kept at −80°C post‐exposure, were used for cytokine analysis after being thawed at 4°C. (a) The bar graph generated from the 23‐plex Luminex assay displays the upregulation of 15 inflammatory cytokines/chemokines following 7‐days CS exposure in C57BL/6J, UNC, and J:DO mouse strains. The analysis was performed using 4–5 mice per group from each strain. Statistical significance was determined using ordinary one‐way ANOVA for multiple comparisons. Levels of significance were indicated as *p < 0.05, **p < 0.01, ***p < 0.001, ****p < 0.0001, ns: Non‐significant compared to the air control group.

**FIGURE 4:**
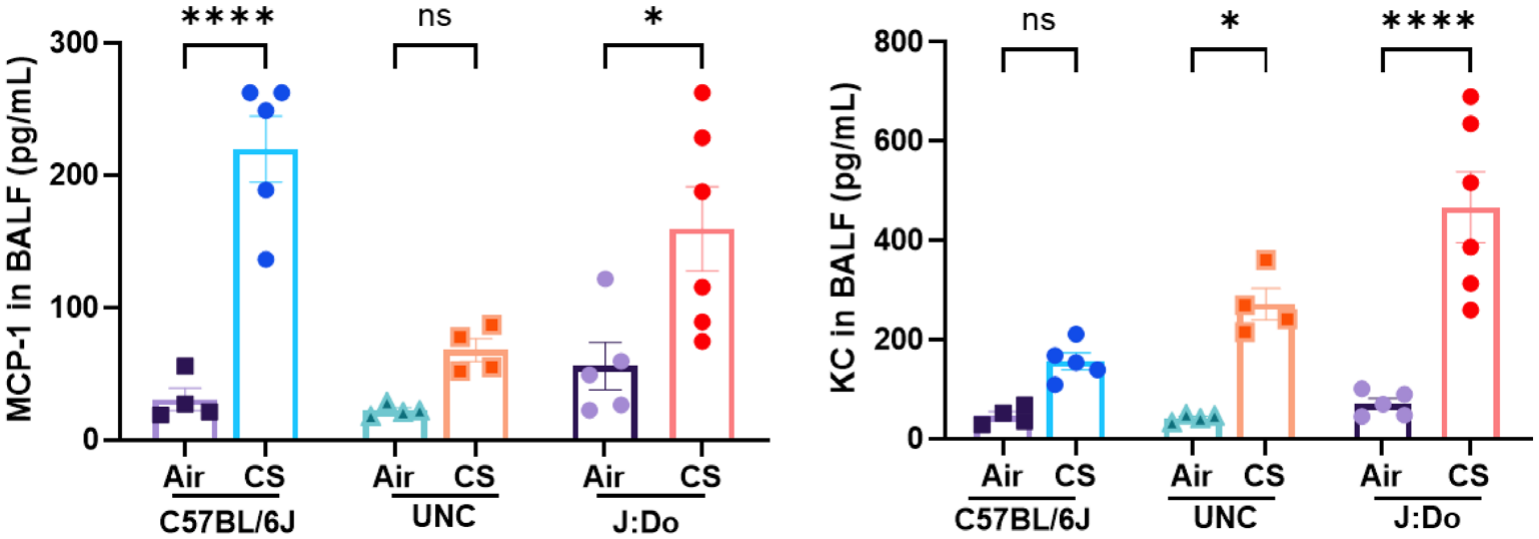
ELISA for KC and MCP‐1 following CS exposure in mouse strains: Bar graphs showing the levels of KC and MCP‐1, derived from single‐plex ELISA, in a 7‐day CS exposure mouse model, *n* = 4–6 mice/group for each strain. Ordinary one‐way ANOVA was employed, followed by Tukey's post hoc test for the analysis. The value of significance was given as: Mean ± SEM. *p < 0.05, ****p < 0.0001, ns: Non‐significant.

Cytokine analysis conducted following CS induction revealed distinct patterns among the strains. After 7 days of mainstream CS exposure, the C57BL/6J strain demonstrated exclusive increased levels of IL‐6, G‐CSF and MCP‐1, the UNC strain showed a predominant upregulation of TNF‐α, IL‐17A and IL‐13, while the J:DO strain displayed elevated KC levels only. Beyond these key cytokines, C57BL/6J mice also displayed significant increases in Eotaxin, IL‐12p40, IL‐1α, Rantes, IL‐10, MIP‐1α, and MIP‐1β, although in varying quantities. Similarly, the UNC strain, in addition to TNF‐α, IL‐17A and IL‐13, showed elevated levels of Eotaxin, IL‐1α, Rantes, IL‐10, IL‐1β, MIP‐1α, and MIP‐1β. The J:DO strain, apart from KC, exhibited upregulation in IL‐12p40, IL‐1α, Rantes, IL‐10, IL‐1β, MIP‐1α, and MIP‐1β (Figure 3). Noteworthily, across all strains, MIP‐1α, MIP‐1β, IL‐1α, IL‐10, and Rantes exhibited a marked increase. Interestingly, when the same 23‐plex cytokine analysis was performed on ETS‐exposed UNC and J:DO mice, no significant changes were observed across all 23 cytokines (Table 1). To further validate the expression of these cytokines, we also conducted single‐plex ELISA for KC & MCP‐1, which confirmed the results from the multiplex assay (Figure 4).

**TABLE 1:** Alterations in cytokine (pg/mL) response in UNC & J:DO Mice following ETS exposure.

Cytokines	UNC AIR	UNC ETS	J:DO AIR	J:DO ETS
Eotaxin	147.07 ± 279.57	1.77 ± 1.69	50.54 ± 115.86	12.88 ± 28.92
G‐CSF	26.70 ± 44.65	15.68 ± 32.77	62.25 ± 148.90	13.02 ± 26.27
GM‐CSF	2.29 ± 0.00	2.30 ± 4.44E‐16	2.29 ± 0.00	2.29 ± 4.44E‐16
IFN‐g	15.83 ± 25.51	5.20 ± 11.09444	46.73 ± 115.46	7.94 ± 13.30
IL‐1a	4.96 ± 9.99	0.03 ± 0.00	63.07 ± 159.64	3.75 ± 8.47
IL‐1b	1.08 ± 0.00	1.08 ± 0.00	1.08 ± 0.00	1.08 ± 0.00
IL‐2	90.43 ± 75.74	86.02 ± 68.50	78.05 ± 157.86	17.20 ± 10.43
IL‐3	0.26 ± 0.00	0.26 ± 0.00	18.61 ± 51.90	0.26 ± 0.00
IL‐4	0.33 ± 0.00	0.33 ± 0.00	0.33 ± 0.00	0.33 ± 0.00
IL‐5	5.67 ± 11.36	2.48 ± 4.84	1.74 ± 2.36	2.23 ± 4.23
IL‐6	0.44 ± 0.00	0.44 ± 0.00	0.44 ± 0.00	0.44 ± 0.00
IL‐9	1.50 ± 0.00	1.50 ± 2.22E‐16	1.50 ± 0.00	1.50 ± 2.22E‐16
IL‐10	99.47 ± 81.32	97.64 ± 78.63	61.47 ± 104.07	19.57 ± 08.37
IL‐12 (p40)	119.03 ± 205.16	7.21 ± 0.00	355.11 ± 852.96	53.60 ± 113.64
IL‐12 (p70)	107.95 ± 204.65	2.32 ± 0.00	413.56 ± 1068.27	38.30 ± 88.13
IL‐13	6.26 ± 8.88E‐16	6.26 ± 8.88E‐16	6.26 ± 8.88E‐16	6.26 ± 8.88E‐16
IL‐17A	5.30 ± 9.55	0.28 ± 0.00	0.637 ± 00.99	1.36 ± 2.63
KC	68.09 ± 56.74	72.11 ± 58.04	33.64 ± 52.80	14.22 ± 06.17
MCP‐1	9.16 ± 1.78E‐15	9.16 ± 1.78E‐15	9.16 ± 1.78E‐15	9.16 ± 1.78E‐15
MIP‐1a	121.64 ± 218.74	0.25 ± 5.55E‐17	38.70 ± 108.76	62.67 ± 152.90
MIP‐1b	344.69 ± 471.85	172.63 ± 136.69	96.49 ± 164.94	34.18 ± 11.00
RANTES	1148.62 ± 2190.61	1.59 ± 2.22E‐16	3917.93 ± 9687.58	478.48 ± 1168.14
TNF‐a	162.80 ± 132.08	183.13 ± 141.16	87.15 ± 137.04	32.41 ± 07.71

### 3.3 Assessment of histological features of different mice strains

**FIGURE 5:**
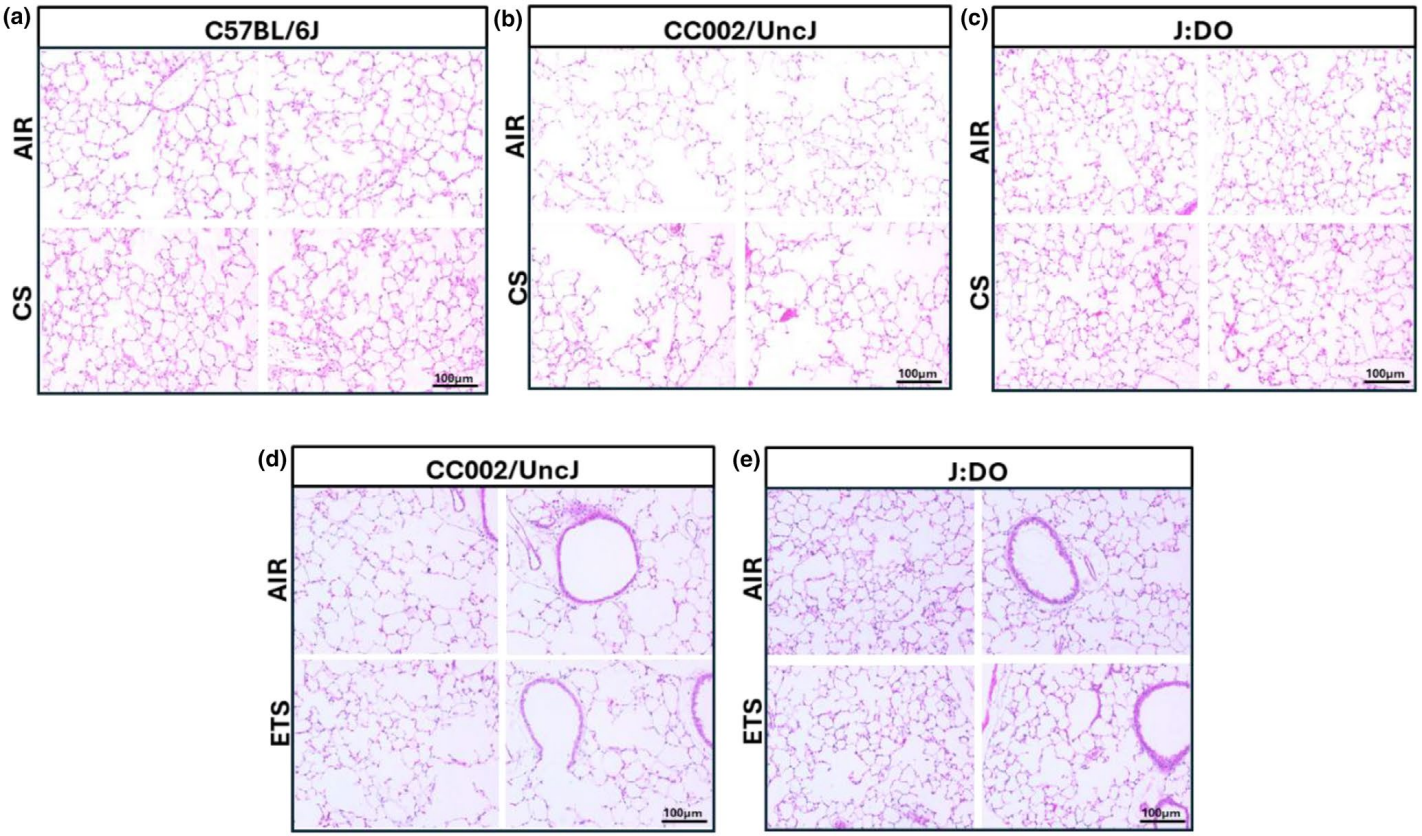
Lung tissue analysis via H&E staining: H&E staining was performed on lung tissues collected post‐exposure. The lungs were first inflated with 1% low‐melting agarose to preserve tissue architecture and then stored in chilled PBS to maintain sample integrity until processing. (a–e) The upper pair of H&E images of each set show the lung tissue architecture (showing alveolar regions) of air‐exposed mice, while the lower pair of each set represent the lungs of mice exposed to CS/ETS including (a) C57BL/6, (b) UNC, and (c) J:DO from the mainstream CS group, and (d) UNC and (e) J:DO from the ETS group. All images were captured with a brightfield microscopy at 20× magnification, with a scale bar of 100 μm.

Morphometric analyses of the lung indicated no signs of airspace enlargement in the lungs of C57BL/6J mice following 7 days CS exposure (Figure 5a). In contrast, the UNC strain was more prone to alterations in alveolar space post 7‐day CS exposure (Figure 5b). It is important to mention here that upon histological assessments the size of the alveolar sac in the tissue sections for the air controls appeared to be larger in UNC as compared to the other two mouse strains. This shows that the inherent size of the alveolar sac in these mice was a bit larger, an area that warrants further work. Finally, the J:DO strain was also assessed for air space enlargement post‐CS exposure, but no observable changes were noted (Figure 5c). In addition to the analysis of lung tissue architecture from mainstream CS exposure, the ETS‐exposed mice strains (UNC and J:DO) showed no changes in the morphological characteristics upon 30‐day ETS exposure (Figure 5d,e). The observed lack of immune cell population within the lungs of UNC mice could be indicative of an inherent lack of alveolar macrophages within the lungs of these mice (section 3.4). In the subsequent section, we aimed to characterize the macrophage mediated inflammatory responses in response to CS in various mouse strains. To confirm their identity, we utilized immunostaining (IHC) to detect CD68‐marker of alveolar macrophages.

### 3.4 Immunohistology of lung tissue for macrophages

**FIGURE 6:**
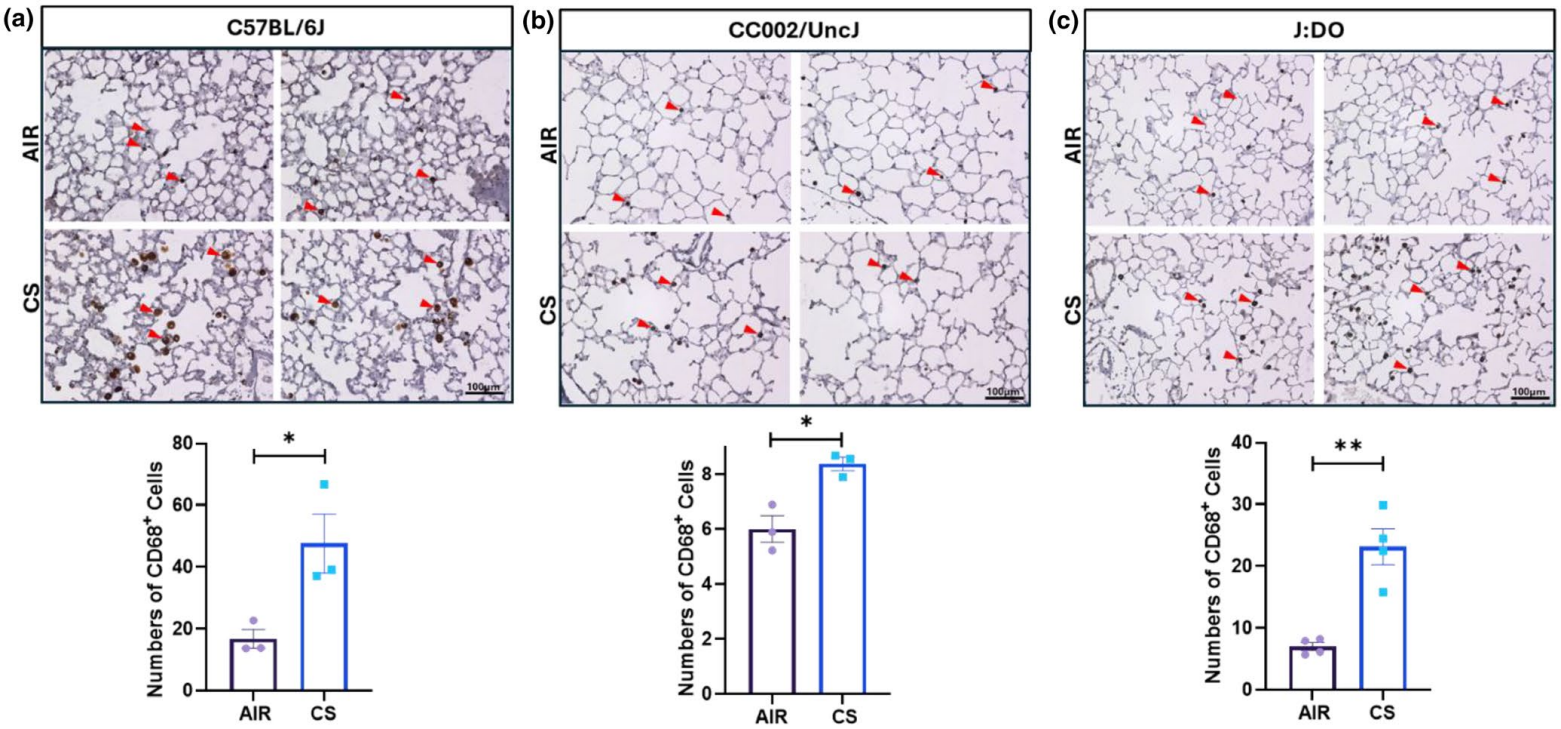
Macrophage cell evaluation in lung tissue via IHC: A 5 μm section of paraffin‐embedded lung tissue from CS exposed mice, which had been inflated with 1% low‐melting agarose, was used for IHC analysis. (a–c) The upper pair of IHC images depict the air group and lower pair of IHC images showed the CS group's alveolar macrophages in dark brown color, indicated by red arrowheads, in the lung tissue of (a) C57BL/6J, (b) UNC, and (c) J:DO mouse strains. (scale bar = 100 μm). The number of macrophages was quantified manually based on CD68+ expression, using a minimum of 10 representative images per sample. Its quantitative data are represented with the bar graphs provided alongside the IHC images, captured with brightfield microscopy at 20X magnification. Student' s t‐test was utilized for the analysis. The value of significance was given as: Mean ± SEM. *p < 0.05, **p < 0.01.

To investigate the alveolar macrophages in the lung tissue sections of mice exposed to mainstream CS, we stained the lung tissue sections with CD68‐antibody (macrophage marker). The IHC images revealed a notable difference in the number of macrophages between air and CS‐exposed mice groups, which was corroborated by quantitative analysis of CD68‐positive cells. In the C57BL/6J strain, there was a significant increase in the CD68+ cells on CS exposure (Figure 6a). This finding aligns with previous reports from our lab indicating that acute CS exposure leads to substantial inflammation in this strain (Yao et al., 2008). The results from the UNC strain were particularly noteworthy. While there was a significant increase in macrophage numbers between the air and CS‐exposed groups, the baseline macrophage count was initially meager, with an approximate rise of 33% only. This may offer a plausible explanation for the enlarged lung airspaces observed in the H&E staining, as a lower initial immune response could contribute to structural changes in lung tissue following exposure (Figure 6b). In the J:DO strain, the number of macrophages was elevated in CS exposed group comparable to its air control (Figure 6c). In response to CS‐induced stress, macrophages secrete various proteins that mediate secretion of inflammatory cytokines and tissue remodeling. Among these, MMP9 and MMP12 play critical roles in degrading the extracellular matrix and regulating inflammatory responses. We specifically targeted the expression of MMP9 and MMP12 in the lungs of 7‐day CS‐exposed mice to assess the severity of CS‐exposure and the activation of macrophages.

### 3.5 Macrophage‐mediated abundance of MMP9 and MMP12

**FIGURE 7:**
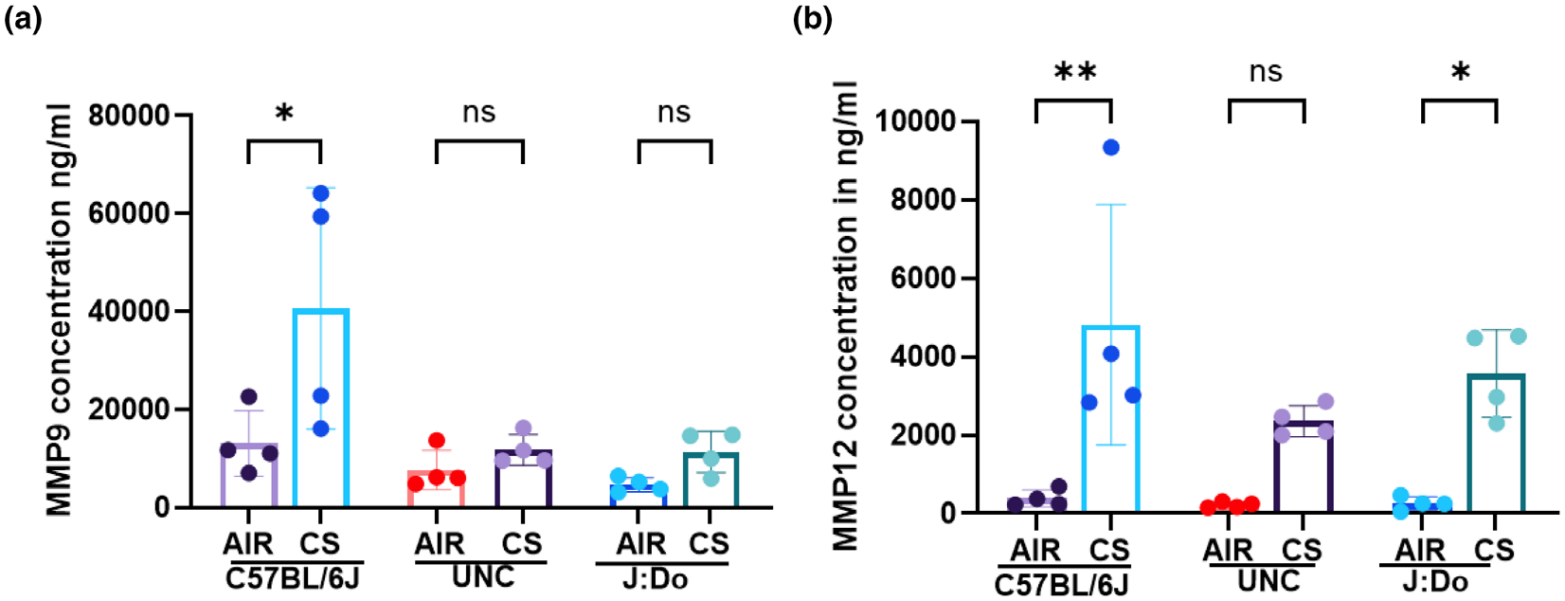
MMP9 and MMP12 abundance in lung tissue homogenate: After the mice were sacrificed, lung tissues from all the groups were stored at −80°C to preserve their integrity. A small portion (~30 mg) of these frozen tissues was subsequently used for protein extraction and preparation for ELISA assays. (a) Bar graph of ELISA results showing MMP9 level in ng/ml of lung tissue homogenate at a 1:50 dilution. (b) Similarly, the bar graph ELISA of MMP12 level in ng/ml, shown at a 1:2 dilution. *N* = 4/group, ordinary one‐way ANOVA was employed, followed by Tukey's post hoc test for the analysis. The value of significance was given as: Mean ± SEM. *p < 0.05, **p < 0.01, ns: Non‐significant.

MMP9 and MMP12 are critical mediators of inflammation and tissue remodeling in response to CS exposure. Their dysregulation plays a crucial role in driving lung damage and inflammation (Cabral‐Pacheco et al., 2020). To investigate the role of these two MMPs in our experimental setup, ELISA was performed on lung tissue homogenates to quantify their levels. The results revealed a significant increased MMP9 level, observed exclusively in the C57BL/6J mice strain. MMP9 is primarily responsible for tissue remodeling and wound healing, so its upregulation likely plays an important role in maintaining lung tissue architecture following exposure, as observed by the H&E images of the C57BL/6J strain (Figure 7a). Regarding MMP12, significant changes were noted across all strains except for the UNC strain (Figure 7b). This aligns with our earlier IHC results, which showed a notably low number of macrophages in the lungs of UNC mice. These findings collectively highlight the strains more susceptible to CS while identifying those that exhibit greater resistance to such exposures and the resulting inflammatory responses.

### 3.6 Comprehensive analysis of inflammation, proinflammatory cytokines, and lung tissue architecture in HET3 mice strains

**FIGURE 8:**
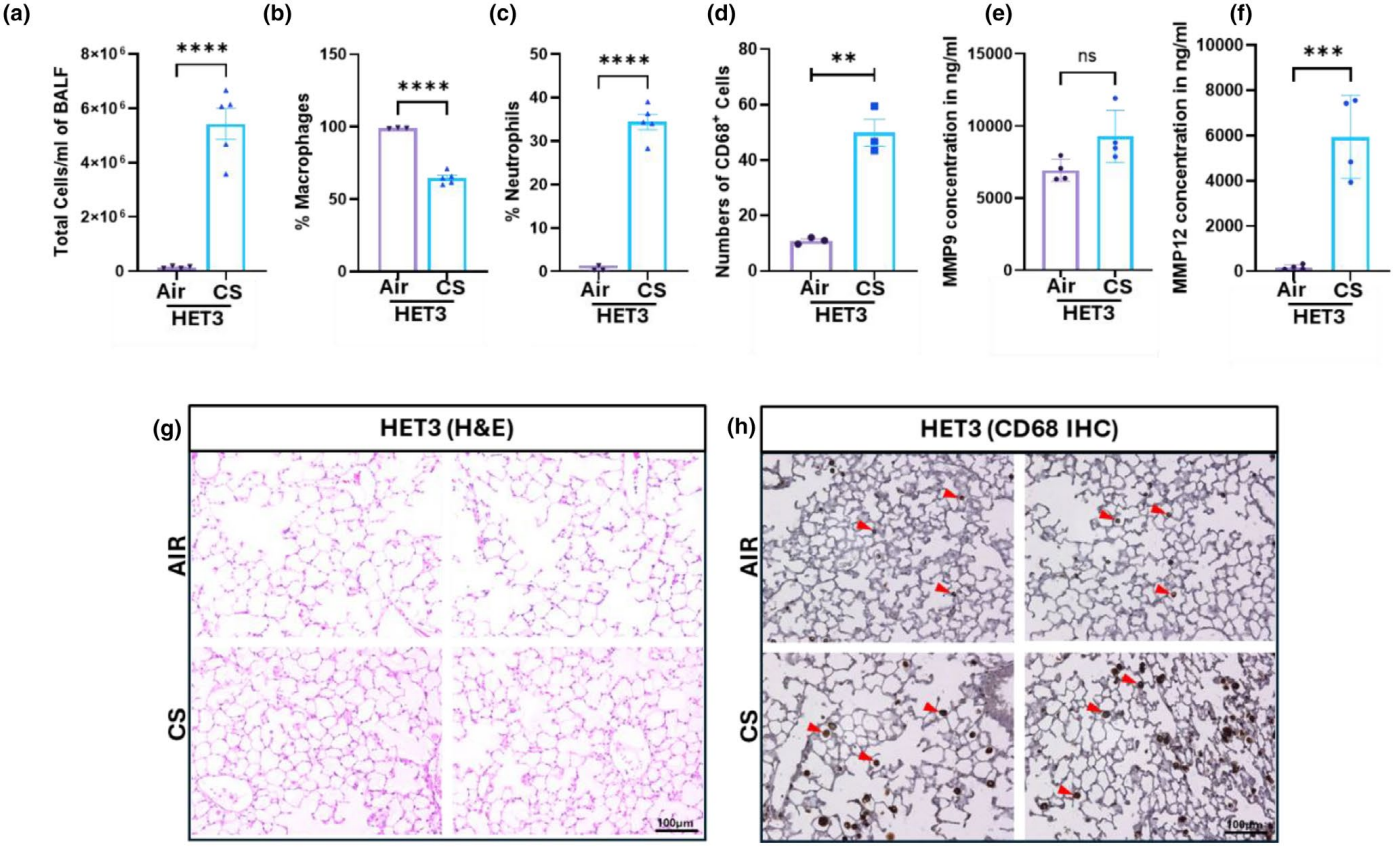
HET3 inflammatory profiling and lung damage assessment following CS exposure: Similar to the previously mentioned mouse model groups, we also collected BALF and lung tissues from HET3 mice post exposure for various analysis, including total cell count and immune cell count to assess inflammatory markers and immune cell infiltration. The lung tissue from these mice groups were further utilized for ELISA, IHC, and H&E staining to assess the tissue architecture changes, macrophages infiltration, MMP9/12 levels. (a) Total cell count/mL of BALF, (b) Percentage Macrophage (c) percentage neutrophil count in the BALF of CS‐exposed HET3 mice after 7 days. (d) Total count of CD68+ cells post‐IHC analysis in air‐ and CS‐exposed groups. Bar graphs of ELISA results for (e) MMP9 and (f) MMP12 levels. (g) H&E images illustrating lung tissue architecture in air‐control and CS‐exposed mice. (h) IHC staining of lung tissue highlighting CD68+ cells (marked with red arrowheads). *N* = 3–5/group, Student's *t*‐test was utilized for the analysis. The value of significance was given as: Mean ± SEM. **p < 0.01, ***p < 0.001, ****p < 0.0001 ns: Non‐significant.

**FIGURE 9:**
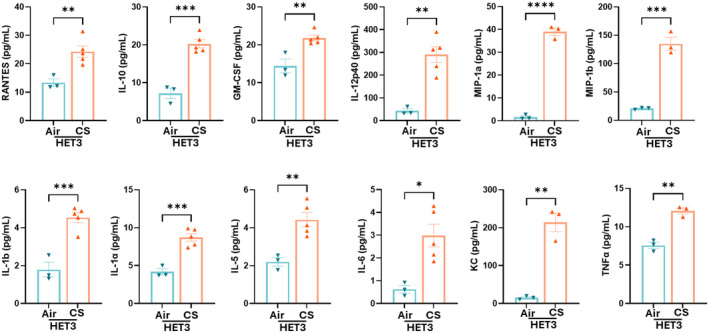
Cytokine analysis of BALF of HET3 mice post 7 days exposure. Bar graphs obtained from the 23‐plex cytokine analysis performed on BALF from air‐ and CS‐exposed HET3 mice groups. *N* = 3–5/group, Student's *t*‐test was utilized for the analysis. The value of significance was given as: Mean ± SEM. *p < 0.05, **p < 0.01 ***p < 0.001, ****p < 0.0001, ns, Non‐significant.

While the initial observations focused on the two genetically diverse mouse strains, here we aimed to investigate the inflammatory response of the HET3 strain, a model typically used for aging‐related studies, following mainstream 7 days CS exposure. To initiate this analysis, we assessed the total cell, macrophage and neutrophil count in the BALF post CS exposure, which revealed a pattern like that observed in C57BL/6J, UNC, and J:DO strains (Figure 8a–c). Further, we evaluated the levels of proinflammatory cytokines in the BALF, which demonstrated a notable upregulation of specific cytokines (MIP‐1α, MIP‐1β, TNFα, Rantes, IL‐10, GM‐CSF, IL‐12p40, IL‐1β, IL‐1α, IL‐5, IL‐6, and KC) post‐exposure. Interestingly, in the HET3 strain, 12 out of the 23 cytokines were notably upregulated (Figure 9). Additionally, GM‐CSF was the only cytokine uniquely elevated in HET3, highlighting its distinct inflammatory response profile compared to other strains. We then extended our analysis to the lung tissue architecture, examining alveolar macrophages following CS exposure. We observed no drastic changes in lung tissue architecture, but a substantial increase in alveolar macrophages in the exposed lung tissue was observed suggesting an active immune response (Figure 8d,g,h). Notably, our analysis of MMP9 expression in the lung tissue homogenates did not reveal significant changes, whereas the MMP12 was highly expressed in CS‐exposed conditions (Figure 8e,f). This also suggests a distinct response to CS exposure in terms of matrix metalloproteinase activity, potentially indicating varying degrees of tissue remodeling or damage between strains.

## 4 DISCUSSION

The genetic diversity in humans makes it difficult to fully understand disease mechanisms when relying on traditional inbred mouse models like BALB/c & C57BL/6J. While these models have been instrumental in studying human diseases for many years, they do not fully replicate the complexity of human biology. The uniform genetic background of these mice limits how applicable the findings are to the genetically varied human population. As a result, insights gained from these mouse models may not always translate effectively to understanding or treating diseases in humans, where genetic and environmental differences play a crucial role in disease progression and treatment response. The introduction of genetically diverse mice, such as the CC and Diversity Outbred (DO) marks a major step forward in overcoming the limitations of traditional inbred mouse models. These mice better capture the genetic variability seen in human populations, allowing researchers to investigate how genetic diversity affects disease risk, progression, and responses to treatment. By using these models, we can not only study the underlying mechanisms of diseases, but also gain insights into how environmental and genetic factors combine to influence disease outcomes. This approach offers a more accurate and realistic avenue to study complex diseases like cancer, cardiovascular diseases, and autoimmune disorders, which often have multifactorial causes in humans (Lampreht Tratar et al., 2018; Nelson et al., 2024; Salimova et al., 2019; Sargent et al., 2022).

The present study investigates the inflammatory response in genetically diverse mice models, specifically J:DO, UNC, and HET3, under stress conditions induced by CS and ETS exposure. While models like J:DO and UNC provide a broader understanding of genetic influences on inflammation, HET3, typically used for age‐related research, offers additional insights (Graham et al., 2024; Harrison et al., 2014; Saul et al., 2019; Soni et al., 2024; Zhu et al., 2022). In contrast, C57BL/6J mice were used as an experimental control, providing parallel findings consistent with prior studies on CS‐induced inflammation. Although prior findings from our lab demonstrated that ETS exposure heightens the risk of severe lung fibrotic responses induced by bleomycin, no significant changes were observed in body weight, airspace enlargement, or collagen deposition in the C57BL/6J mice subjected to ETS exposure (Wang et al., 2024).

Previous studies have established the importance of using genetically diverse mice to explore CS‐induced inflammatory responses (Easwaran et al., 2022; Feller et al., 2018; Huvenne et al., 2010). However, few studies have examined the inflammatory response to CS and ETS simultaneously (Yao et al., 2008). Our findings expand on this by showing a general increase in total cell numbers across all strains post‐CS/ETS exposure. Furthermore, the results reveal unique patterns in macrophage and neutrophil counts. While neutrophils showed a notable increase across all strains, the variation in macrophage numbers was more subtle but still significant. One striking finding was the notably low macrophage count in the airspace of the UNC strain.

The primary goal of this study was to investigate cytokine regulation linked to acute CS/ETS exposure. Initial analyses targeted cytokines secreted by macrophages and neutrophils, such as IL‐6, TNF‐α, MCP‐1 and KC. Interestingly, we did not observe consistent upsurge of these cytokines across all strains. However, other cytokines, including MIP‐1α, MIP‐1β, IL‐1α, IL‐10, and Rantes, showed significant upregulation across all strains. MIP‐1α and MIP‐1β are reported to be a key player in recruiting macrophages and T‐cells to the site of infection, emphasizing their role in inflammation (Chen et al., 2023; Scapini et al., 2000). IL‐10, produced by macrophages in various immune contexts, also plays an essential role in controlling immune‐mediated pathology (Sanin et al., 2015). Whereas IL‐1 is a major proinflammatory cytokine derived from macrophages, functioning primarily by triggering a cascade of cytokines, chemokines, and small molecule mediators (Carmi et al., 2009). Interestingly, despite having fewer macrophages and larger airspaces, the UNC strain exhibited a significant increase in both IL‐10 and IL‐1α levels. An elevated level of Rantes throughout the all the strains signifies the severity of inflammation, as it plays a key role in attracting immune cells from peripheral blood to the site of inflammation (Mikolajczyk et al., 2016).

Further analysis revealed that after mainstream CS exposure, certain cytokines were selectively enhanced in specific strains. For example, TNFα, IL‐17A and IL‐13 expression were increased only in the UNC strain, whereas the KC was elevated in J:DO. MCP‐1, IL‐6 and G‐CSF were exclusively upregulated in the C57BL/6J strain and GM‐CSF was heightened only in HET3 strain. In contrast, the cytokine levels in the ETS groups remained unchanged, correlating with the observation that lung tissue architecture was largely undamaged in these groups. The absence of the C57BL/6J mouse strain in the ETS exposure group may limit our ability to observe clear differences, as seen with mainstream smoke exposure. However, our primary goal is to gain insights into the responses of genetically diverse mice strains. Therefore, we have focused exclusively on these two strains for the ETS exposure analysis.

When examining lung tissue structure, we observed that the C57BL/6J and J:DO strains did not exhibit significant alveolar space enlargement. However, the UNC strain was susceptible to air space enlargement, with a significantly low baseline of alveolar macrophages. Despite the differences in macrophage counts, these variations were still significant across all strains. Macrophage‐mediated MMPs play a crucial role in the inflammatory process. It has been reported that macrophage migration during inflammation is facilitated by the activation of MMP‐9 (Gong et al., 2008). MMP9 is also essential for macrophage fusion and contributes to the function of foreign body giant cells (MacLauchlan et al., 2009). Whereas the MMP12, secreted by alveolar macrophages, is involved in extracellular matrix remodeling, a critical component of physiological tissue repair (Noël et al., 2021). Additionally, MMP12 can also degrade the basement membrane, enabling macrophages to access wounded tissue, and inactivates the complement cascade, which typically attracts inflammatory cells during injury (Lin et al., 2023; Mouton et al., 2018). In our experimental setup, MMP‐9 and MMP‐12 expression analysis revealed distinct patterns across the strains. MMP‐9 was significantly elevated in C57BL/6J mice, but showed no change in the UNC and J:DO strains. On the other hand, MMP‐12 was highly expressed in C57BL/6J, HET3 and J:DO but not in the UNC strain, potentially providing the evidence of a lower macrophage count in the UNC strain. These findings suggest that differences in matrix metalloproteinase activity may contribute to varying degrees of tissue remodeling and susceptibility to lung damage across the different strains.

So far, our findings suggest that the J:DO strain, similar to the C57BL/6J strain, showed a particularly strong macrophage and neutrophil driven inflammatory response and MMP12 activity. This indicates that the J:DO strain might be a suitable model for studying inflammation in genetically diverse mice, particularly when both macrophages and neutrophils are involved. On the other hand, the UNC strain, although it demonstrated a strong neutrophilic response, might not be a suitable strain due to its low baseline macrophage activity, likely because of an inherent deficiency in macrophages (possibly lower monocyte/macrophages pool and release by bone morrow or lower recruitment in interstitial areas), warranting further studies to explain this difference carefully. Additionally, an investigation on UNC mice revealed an exaggerated eosinophilic response and asthma phenotypes, including airway hyperresponsiveness, after repeated ozone (O_3_) exposure. This study offers a valuable evidence for investigating mechanisms linking O_3_ exposure to asthma using UNC mice strain (Smith et al., 2021). Although C57BL/6J was used as a control, it is important to recognize that it may not be the ideal control for a study of this nature. Moreover, the small sample size restricted our ability to explore potential sex‐based variations, an area of significant interest for future research. Additionally, the short duration of exposure may not accurately reflect the progression of disease, limiting our ability to capture the full spectrum of CS‐induced effects.

In summary, this study highlights the importance of using genetically diverse mouse models to better understand the inflammatory response to CS/ETS exposure. The observed variations in cytokine profiles, immune cell counts, and lung tissue architecture provide valuable insights into the complex interactions between genetics, inflammation, and tissue damage.

## AUTHOR CONTRIBUTIONS

M.I.F. contributed to manuscript writing, data procurement, and data analysis. G.K. offered valuable suggestions during the study design, assisted with data analysis, provided key input during the experimental phase and contributed to proofreading the manuscript. S.S. was instrumental in helping with mouse exposures and ELISA assays and contributed to proofreading the manuscript. F.E. provided support with immunohistochemistry and assisted in the proofreading process. H.U. provided funding and conducted the final review. I.R. secured funding, provided critical guidance on the study design, and did a final review of the manuscript.

## FUNDING INFORMATION

This study was supported by the National Institutes of Health (NIH) R01 ES029177, R01 HL147715, RO1 HL158316 and RO1 HL167655. The funding body has no role in design of the study, data collection, analysis, interpretation of data, or in writing the manuscript.

## CONFLICT OF INTEREST STATEMENT

The authors have declared no competing interest.

## Data Availability

All data and materials are described in the manuscript and will be made available upon request.
